# Repeat-encoded poly-Q tracts show statistical commonalities across species

**DOI:** 10.1186/1471-2164-14-76

**Published:** 2013-02-02

**Authors:** Kai Willadsen, Minh Duc Cao, Janet Wiles, Sureshkumar Balasubramanian, Mikael Bodén

**Affiliations:** 1 School of Chemistry and Molecular Biosciences, The University of Queensland, Brisbane QLD 4072, Australia; 2 School of Information Technology and Electrical Engineering, The University of Queensland, Brisbane QLD 4072, Australia; 3 School of Biological Sciences, Monash University, Victoria 3800, Australia

## Abstract

**Background:**

Among repetitive genomic sequence, the class of tri-nucleotide repeats has received much attention due to their association with human diseases. Tri-nucleotide repeat diseases are caused by excessive sequence length variability; diseases such as Huntington’s disease and Fragile X syndrome are tied to an increase in the number of repeat units in a tract. Motivated by the recent discovery of a tri-nucleotide repeat associated genetic defect in *Arabidopsis thaliana*, this study takes a cross-species approach to investigating these repeat tracts, with the goal of using commonalities between species to identify potential disease-related properties.

**Results:**

We find that statistical enrichment in regulatory function associations for coding region repeats – previously observed in human – is consistent across multiple organisms. By distinguishing between homo-amino acid tracts that are encoded by tri-nucleotide repeats, and those encoded by varying codons, we show that amino acid repeats – not tri-nucleotide repeats – fully explain these regulatory associations. Using this same separation between repeat- and non-repeat-encoded homo-amino acid tracts, we show that poly-glutamine tracts are disproportionately encoded by tri-nucleotide repeats, and those tracts that are encoded by tri-nucleotide repeats are also significantly longer; these results are consistent across multiple species.

**Conclusion:**

These findings establish similarities in tri-nucleotide repeats across species at the level of protein functionality and protein sequence. The tendency of tri-nucleotide repeats to encode longer poly-glutamine tracts indicates a link with the poly-glutamine repeat diseases. The cross-species nature of this tendency suggests that unknown repeat diseases are yet to be uncovered in other species. Future discoveries of new non-human repeat associated defects may provide the breadth of information needed to unravel the mechanisms that underpin this class of human disease.

## Background

Repetitive sequences are ubiquitous within eukaryotic genomes. While in some contexts these sequences are ignored, for example to avoid false positives when searching sequence databases [1], repetitive DNA tracts are not isolated to intergenic regions; repeat tracts also occur within genes and promoter regions, and length variability in some tracts has known phenotypic effects, including morphological variation in dogs [2] and strength of cell surface adhesion in yeast [3]. Repeat tracts are unstable (i.e., have high mutation rates) in comparison with non-repetitive DNA [4], and the degree of instability varies widely between tracts.

Short tandem repeat tracts can be classified by length of the repeat unit; tracts where the repeated unit is up to six nucleotides long are referred to as microsatellites, with repeats consisting of longer units being referred to as minisatellites. A particular subset of microsatellites – those consisting of a repetitive three-base-pair unit, called tri-nucleotide repeats (TNRs) – have been the focus of much study due to their association with an important class of human diseases, commonly referred to as tri-nucleotide or triplet repeat disorders. Around thirty TNR diseases such as Huntington’s disease (a coding region repeat) and Friedreich’s ataxia (an intronic repeat) have been identified [5]. Such diseases are caused by variation in the number of copies of the repeated sequence – most commonly expansion, though contraction diseases also exist. Many of these diseases affect the nervous system, and demonstrate genetic anticipation; that is, as the copy number of the repeat sequence diverges from the population norm, the age of onset decreases while symptoms increase in severity [6].

While the exact causes of excessive variability in a specific repeat tract remain an open question, several features are generally agreed to contribute to high variability of repetitive sequences: length (i.e., number of repeats), purity (i.e., number of interruptions to the repetitive pattern) and sequence (i.e., the nucleotide sequence being repeated) [7,8]. However, these features are not sufficient to determine expansion; flanking sequences [9] and repeat orientation with respect to origin of replication initiation [10] have been shown to be factors affecting whether repeats will undergo expansion.

The prevalence of these repetitive tracts and their distinctive characteristics have made large-scale surveys an appealing avenue for identifying potentially useful features for explaining their variability [11,12]. Such surveys have focussed on TNRs in the human genome, likely due to both the availability of data and the relevance to understanding repeat diseases.

Until recently, all characterised TNR diseases were human-specific, but the recent discovery of a TNR mediated genetic defect in *Arabidopsis thaliana*[13] supports the idea that both the mechanisms and the underlying causes are cross-species phenomena. This discovery raises questions about whether there are cross-species commonalities between repetitive sequences – specifically TNRs – that may help us to understand what makes a specific TNR sequence prone to repeat number instability, and the diseases that can result. Identification of naturally-occurring TNR diseases in other organisms also expands the scope of possible study in those model organisms. (For a summary of model systems and their characteristics for TNR study, see supplementary information in [5].)

One identified characteristic of human TNR sequences is that genes containing these repeats – and more specifically repeats in coding regions – have been shown to be significantly associated with regulatory function through gene ontology (GO) term analyses [12]. Given the increased instability of TNR tracts, do these sequences have specific properties that support or enable regulatory function? For example, similar regulatory function associations have been observed in proteins containing repetitive homo-amino acid (homo-AA) tracts [14,15], a likely product of exonic TNRs. These observations raise the question of whether TNR sequences’ functional associations are properties of the repeat sequences themselves, or whether the observed associations can be explained by repetitive amino-acid tracts in the resulting proteins.

In this study, we investigate the functional associations of TNR sequences across different species to see whether cross-species analyses support the purported functional roles of repetitive sequences, and whether these functional roles can be explained by sequence properties common to multiple species. In particular, we ask whether the functionality of TNR sequences in multiple species can be explained by their associated proteins’ amino acid repeat tracts. Identifying the functional roles of existing TNR sequences is a crucial step in understanding repeat variability, and expanding such knowledge across multiple species provides valuable background knowledge in selecting model organisms for studying the mechanisms of repeat variability.

## Results

### Cross-species occurrence of tri-nucleotide repeats

As a first step towards understanding species-specific characteristics of TNRs, we identify and analyse TNR tracts in six different species: *Saccharomyces cerevisiae*, *Arabidopsis thaliana*, *Caenorhabditis elegans*, *Drosophila melanogaster*, *Mus musculus* and *Homo sapiens*. Repeat tract scanning identified 247, 1947, 559, 3996, 79727 and 35736 TNR sequences in these species, respectively. As repeat length and repeat sequence are widely accepted factors in TNR variability, we compare these properties across organisms.

**Figure 1:**
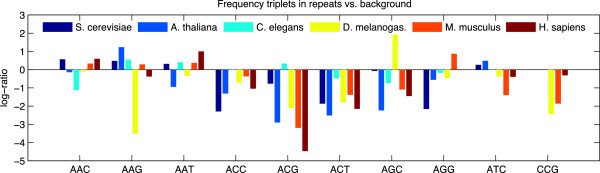
**Ratio of TNR sequence frequencies to genomic background.** Differences shown are the log-ratio of the frequency of TNRs with the specific sequences identified vs. whole-genome order-two Markov backgrounds. TNR sequence frequencies vary markedly across different organisms. In all organisms, TNR sequence distribution was very different from the background, and organisms also have very different distributions from one another.

Length distribution of triplets was broadly similar across species (see Additional file 1: Figure S1). However, comparison of the distribution of repeat unit sequences showed more interesting patterns (see Figure 1). In all organisms, distribution of sequences in identified TNR tracts was significantly different to the background triplet frequency (*p*≪1e^−14^ for all species, using a chi-squared test against a genome-wide order-two Markov background), agreeing with earlier genome-wide analyses of human repeats [12]. More interestingly, the distribution of triplet sequences in different organisms demonstrated markedly different patterns of non-randomness, even after compensating for different backgrounds (i.e., using per-organism and per-chromosome backgrounds, comparing log-ratios across organisms).

It is interesting to note the large differences in TNR frequencies among these genomes. Notably, the *Drosophila melanogaster* genome (∼165Mb) contains more than six times as many TNR sequences as the *Caenorhabditis elegans* genome (∼100Mb); there are over twice as many TNRs in the mouse genome than in the human genome. The latter is particularly striking since their genomes are similar in size and the large degree of homology between them.

These analyses suggest that simple uses of known correlates of TNR expansion are unlikely to produce informative cross-species patterns. While the differing distribution of triplet sequences may be curious, it does not provide any new insights into the structural characteristics or function of TNR sequences. As an alternative approach, we focus on higher-level characteristics such as the known functional associations and the homo-AA tract composition of TNR sequences.

### Cross-species functionality of tri-nucleotide repeat sequences

**Figure 2:**
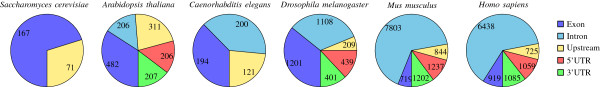
**Identification and division of tri-nucleotide repeats in coding regions.** Tri-nucleotide repeats in coding regions were identified from genomic scans using Tandem Repeat Finder (see Methods for details). The TNRs are then localised to coding regions according to genomic feature from RefSeq annotations.

Human gene-associated repeats – particularly coding region repeats – are known to have overrepresented GO terms indicative of regulatory function [12]. To test whether these functional associations are a cross-species phenomenon, we performed an analysis on the mentioned species. We isolated TNR sequences in these species’ genomes that could be localised to coding regions according to genomic feature annotations (See Figure 2). This set of repeat-containing genes were then analysed for systematic over-representation of GO terms. We found that, in several species, TNR-containing coding sequences showed over-represented regulation-associated GO terms (see Table 1), supporting a view that these associations are a cross-species phenomenon.

**Table 1:** Top-5 over-represented GO/Biological process terms in exonic repeat-associated genes by species

***Saccharomyces cerevisiae***		
GO term	E-value	Term description
GO:0050789	2.35E-06	regulation of biological process
GO:0060255	8.91E-06	regulation of macromolecule metabolic process
GO:0050794	9.14E-06	regulation of cellular process
GO:0019222	9.40E-06	regulation of metabolic process
GO:0048522	1.49E-05	positive regulation of cellular process
***Arabidopsis thaliana***		
GO term	E-value	Term description
GO:0016070	7.32E-10	RNA metabolic process
GO:0090304	1.59E-09	nucleic acid metabolic process
GO:0044260	1.78E-09	cellular macromolecule metabolic process
GO:0009889	2.61E-09	regulation of biosynthetic process
GO:0043170	2.74E-09	macromolecule metabolic process
***Caenorhabditis elegans***		
GO term	E-value	Term description
GO:0007265	2.22E-08	Ras protein signal transduction
GO:0046578	7.32E-08	regulation of Ras protein signal transduction
GO:0050794	1.29E-07	regulation of cellular process
GO:0009966	5.92E-07	regulation of signal transduction
GO:0051056	1.11E-06	regulation of small GTPase mediated signal transduction
***Drosophila melanogaster***		
GO term	E-value	Term description
GO:0048856	5.57E-106	anatomical structure development
GO:0048731	3.19E-100	system development
GO:0007275	8.55E-95	multicellular organismal development
GO:0032502	9.65E-95	developmental process
GO:0048513	1.10E-90	organ development
***Mus musculus***		
GO term	E-value	Term description
GO:0032502	3.76E-45	developmental process
GO:0007399	4.92E-42	nervous system development
GO:0007275	2.98E-41	multicellular organismal development
GO:0048856	2.61E-39	anatomical structure development
GO:0048869	3.83E-39	cellular developmental process
***Homo sapiens***		
GO term	E-value	Term description
GO:0007399	1.15E-20	nervous system development
GO:0030030	5.48E-16	cell projection organization
GO:0032989	6.39E-16	cellular component morphogenesis
GO:0048666	2.81E-15	neuron development
GO:0000902	3.76E-15	cell morphogenesis

Note that for *Drosophila melanogaster*, *Mus musculus* and *Homo sapiens*, the top-5 terms are development-related, yet many regulation-related terms appear at statistically significant levels (not shown).

From these results we conclude that the previously-observed regulation association of coding region TNRs is not exclusively a human-specific trait, but can be seen as a cross-species phenomenon, even across a range of dissimilar organisms. Importantly, these results do not address the possibility that the functional associations are the result of a derivative sequence property, such as the homo-amino acid repeat tracts in corresponding proteins.

### Homo-amino acid repeat sequences and tri-nucleotide repeats

Previous GO over-representation analyses of TNR se- quences have used whole organism gene sets as a statistical background [12]. However, when looking at functional associations of genes containing coding sequence-localised TNR tracts, it must be noted that the protein sequences associated with these genes will constitute an unusual subset of the proteome, and may give a very different statistical background. Specifically, translations of TNR tracts will result in protein sequences enriched in homo-AA tracts.

Through their association with TNRs, protein homo-AA repeat tracts have been implicated in a range of human diseases [16] and are more likely to be involved in transcriptional regulation [15], possibly due to the structural characteristics of the homo-AA tract. It has been suggested that these tracts are inherently structurally disordered [17-19], and that such unstructured regions may act as flexible regions, increasing binding affinity [18]. The prevalence of transcription factors amongst homo-AA repeat-containing proteins raises the question of whether functional associations previously ascribed to coding sequence TNRs may be explained by the homo-AA tracts they encode.

Due to the redundancy of the genetic code, a homo-AA tract is not necessarily encoded by a TNR tract; instead, variant encodings may be used and in fact, they are expected to be less prone to mutation-driven length variability. As such, repetitive DNA sequence encoding important regulatory functional elements appears less than optimal, unless there is a associated functional difference.

In order to identify the degree to which homo-AA tracts in TNR-associated proteins explain the functional associations of these nucleotide repeats, we performed whole-proteome repeat scans of each organism’s non-redundant proteome and split the identified homo-AA tract containing proteins (hereafter simply referred to as homo-AA proteins) into two sets – those where the homo-AA tract was encoded by a repetitive DNA sequence, and those where variant encoding was in use – before identifying functional associations of these sets using GO terms.

GO term over-representation testing of the TNR-encoded homo-AA proteins was initially done using the variant-encoded homo-AA protein set as a background model; this test identifies whether the TNR-encoded set is significantly different from the variant-encoded set. We also used the whole set of homo-AA containing proteins as a background, identifying whether TNR-encoded proteins form an identifiably distinct subset of all homo-AA proteins in terms of functional associations.

Our analysis identified 299, 1285, 892, 2252, 1530 and 1661 homo-AA proteins in *Saccharomyces cerevisiae*, *Arabidopsis thaliana*, *Caenorhabditis elegans*, *Drosophila*, mouse and human respectively, including 96, 337, 67, 404, 253 and 342 proteins containing TNR-encoded homo-AA tracts (see Table 2). GO over-representation analysis of TNR-encoded homo-AA proteins revealed very similar results to those produced for coding region TNRs (data not shown), suggesting that homo-AA associated TNRs are a representative subset of all coding region TNRs.

**Table 2:** Division of TNR- and variant-encoded homo-AA proteins

	**TNR-encoded**	**Variant-encoded**	**All**
**Species**	**homo-AA proteins**	**homo-AA proteins**	**homo-AA proteins**
*Saccharomyces cerevisiae*	96	224	299
*Arabidopsis thaliana*	337	985	1285
*Caenorhabditis*	67	834	892
*Drosophila melanogaster*	404	2083	2252
*Mus musculus*	253	1369	1530
*Homo sapiens*	342	1416	1661

Note that a protein may contain both TNR- and variant-encoded homo-AA tracts. The number TNR-encoded proteins may be lower than the number of TNR tracts in exonic regions because a stricter criterion was applied to determine TNR-encoded homo-AA tracts, which did not allow for interruptions.

In *Saccharomyces cerevisiae*, *Arabidopsis thaliana* and human, TNR-encoded homo-AA proteins show no over-represented GO terms when the variant-encoded set is used as background. A few over-represented terms in *Caenorhabditis elegans*, *Drosophila melanogaster* and mouse remain (weak, and largely development-associated, see Table 3) but no regulatory associations are identified. Significantly, these findings show that the previously-observed regulatory associations of exonic TNRs are explained by the function of the homo-AA tracts they encode, controverting existing notions of the roles undertaken by these tracts. As such, any attempt to identify a role for TNRs occurring in coding sequence should focus on other characteristics distinguishing proteins encoded by TNR sequences.

**Table 3:** Over-represented GO terms in TNR encoded homo-AA tract containing proteins

**Species**	**GO term**	**E-value**	**Term description**
*Caenorhabditis elegans*	GO:0006996	2.90E-02	organelle organization
*Drosophila melanogaster*	GO:0005917	1.45E-02	nephrocyte diaphragm
	GO:0034333	1.45E-02	adherens junction assembly
	GO:0036058	1.45E-02	filtration diaphragm assembly
	GO:0036059	1.45E-02	nephrocyte diaphragm assembly
	GO:0036056	1.45E-02	filtration diaphragm
*Mus musculus*	GO:0051276	3.09E-02	chromosome organization

All over-represented GO terms in TNR-encoded homo-AA tract-containing proteins found in all species when using all homo-AA proteins as a statistical background. All p-values given are Bonferroni-corrected.

### Homo-amino acid tracts and sequence stability

Increased sequence instability is a distinguishing feature of TNR tracts as a whole; in TNR-encoded protein-coding repeat regions, such instability underlies the repeat diseases, but may also affect other aspects of protein function, such as the number and type of interactions the encoded protein is involved in. However, similar instability would not be expected in variant-encoded tracts. In order to identify whether sequence instability is a distinguishing factor of TNR-encoded homo-AA tract proteins, we investigated two characteristics related to sequence stability: protein-protein interaction (PPI) counts and estimated sequence conservation.

A protein’s number of PPIs and its evolutionary rate have been shown to be linked; it has been observed that proteins with higher PPIs evolve more slowly, likely due to sequence constraints involved in maintaining existing interactions [20], though other factors such as expression levels also contribute [21]. As such, the higher variability commonly associated with TNR tracts suggests that homo-AA proteins should have lower PPI counts than their variant-encoded counterparts.

Using the same TNR- vs. variant-encoded distinction as above, and PPI data from the IntAct database, we identified the number of PPIs each homo-AA protein is involved in. Looking at the distribution of PPI counts in these proteins (see Additional file 2: Figure S2) we find that there is no significant difference between TNR- and variant-encoded homo-AA proteins in terms of the number of protein interactions associated with each set.

A different approach to investigating sequence stability is to directly assess the conservation of the homo-AA encoding sequence itself. We used pre-computed *Drosophila* and human PhastCons scores from UCSC (see Methods) to evaluate sequence-level conservation. Genomic loci corresponding to homo-AA encoding regions were obtained by reverse-mapping homo-AA tract boundaries onto exonic sequence. From these loci and PhastCons scores, we obtained conservation metrics for individual tracts. Segmenting these conservation scores as above, we found that conservation of homo-AA encoding DNA was not significantly affected by whether the sequence was classified as a TNR.

Note that this finding does not contradict previous findings that TNR sequences show higher variability. The comparison here is with variant-encoded homo-AA sequences, which constitute a very specific background model. In addition, PhastCons scores are not well-suited to identifying repeat length variation; as such, this method will not account for a major factor in the variability of TNR tracts.

### Homo-amino acid tract composition

As repeat unit and repeat length are central factors in determining TNR variability, considering these factors is also essential when investigating homo-AA proteins. Using the same TNR- and variant-encoded protein sets as above, we classified homo-AA proteins by the repeated residue and compared the frequency and length of residues between the sets. The hypothesis was that there would be no difference between the TNR- and variant-encoded sets in terms of amino acid make-up of repeat regions.

**Figure 3:**
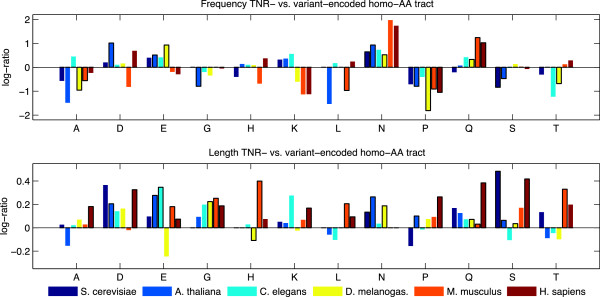
**TNR- vs. variant-encoded amino acid repeats in multiple organisms.** Top: The log-ratio of the proportion of amino acid repeats for TNR- vs. variant-encoded tracts. Bottom: The log-ratio of the length of amino acid repeats for TNR- vs. variant-encoded tracts. Significant (*p*<0.05) differences are identified by bars with a black outline.

Looking at residue frequencies, we found that for human, mouse and fly, glutamine repeats were significantly more likely to be encoded by TNRs than by variant encoding while proline was significantly less likely to be TNR-encoded (see Figure 3). TNR-encoded tracts also tend to be more prevalent to code for glutamic acid and asparagine repeats in most species. In terms of length, we found that in all organisms, the average TNR-encoded poly-glutamine repeat tracts were longer than in their variant-encoded counterparts; the results were significant for human, mouse and fly (see Figure 3); for human only, homo-AA tracts of a number of other amino acids (alanine, aspartic acid, glutamic acid, glycine, lysine, leucine, proline and serine) were also significantly longer in TNR-encoded homo-AA proteins (see Additional file 3: Tables S1 and Additional file 4: Table S2 for more detail). These findings show that poly-glutamine tracts are notably different when encoded by TNRs, and that the differences are consistent with characteristics of human repeat disease, as discussed below.

## Discussion

These analyses have demonstrated that there is evidence for a link between coding sequence TNRs and regulatory function, and that this link is not unique to humans, but can also be seen to different degrees in other species. However, we have also shown that these functional associations – previously characterised for human exonic sequences [12] – are entirely explainable in terms of the characteristics of the resulting proteins, and specifically the homo-amino acid tracts encoded by these sequences. Furthermore, few additional associations were found for either TNR- or variant-encoded homo-AA proteins, suggesting that the increased variability typically associated with tri-nucleotide repeat sequences appears to be neither a benefit nor a barrier in considering functional aspects of the resulting gene products.

While these findings do not contradict the suggestion that expanded exonic tandem repeat regions may be co-opted to fulfil a functional role as regulation-enhancing or -enabling structures, they do strongly suggest that there is nothing functionally unique about TNR sequences. Instead, we suggest that the strong GO term associations previously attributed to TNR tracts are indicative of opportunistic use of existing repeat sequences, a position supported by the cross-species nature of the associations observed above.

Relevant questions have been raised concerning why high-purity homo-AA tracts are so prevalent within structurally disordered proteins, given that repetitive tracts are not necessary for encoding disordered regions [19]. One hypothesis is that high purity in amino acid repeats reflects evolutionary recency in underlying TNRs, driven by microsatellite proliferation and expansion processes [19]. Our study indicates that there is no clear evidence for this hypothesis: no significant difference was found between the number of protein-protein interactions – used here as a proxy for evolutionary constraints – between TNR- and variant-encoded homo-AA proteins, and the nucleotide-level conservation of homo-AA-encoding exonic tracts was likewise unaffected by encoding distinctions. In addition, less than a third of homo-AA proteins could be directly linked to TNR encoding in any organism. These results suggest that evolutionary recency or other TNR-derived properties provide little explanation for the prevalence of pure repeats in structurally disordered proteins.

In contrast to the above results that discount observed or theorised TNR associations, our analysis of homo-AA tract composition shows striking differences between tracts that are TNR- and variant-encoded. In all organisms studied except *Saccharomyces cerevisiae*, glutamine repeats were more likely to be encoded by a TNR sequence than by a variant encoding; for all species, these repeats were also longer when repeat-encoded. This abundance of glutamine repeats among TNR-encoded homo-AA repeat tracts suggests that a correspondence may be drawn with the prevalence of poly-glutamine diseases among known human TNR diseases [16]. Glutamine-encoding CAG ·CTG repeats have been the focus of much research due to their disease associations, and here we show that TNR-encoding of glutamine repeats is associated with longer repeat tracts, without taking into account any disease associations. In addition, this pattern is evident in multiple organisms with no currently characterised poly-glutamine diseases. In combination with the recent characterisation of a TNR-associated genetic defect in *Arabidopsis*[13], this finding supports the notion that poly-glutamine and other protein repeat diseases may be found in non-human contexts, which would provide a wider range of model organisms for studying the mechanisms and determinants of repeat disease.

## Conclusion

By taking a cross-species approach linking homo-amino acid repeat tracts in proteins with tri-nucleotide repeats, this study has explained the regulatory function associations seen among TNR-containing genes. Analysing homo-AA tract-containing proteins, we identified cross-species commonalities in TNR-encoded protein repeat tracts; specifically, that TNR-encoded poly-glutamine repeats show several consistent cross-species statistical patterns. These results raise questions about the existence of undiscovered repeat mediated phenotypes in other species, and whether such repeats may share a broader cross-species statistical profile.

## Methods

### Genomic data

The human (hg19), mouse (mm10), *Drosophila melanog- aster* (dm3) and *Caenorhabditis elegans* (ce10) reference genomes and genomic feature locations were obtained from the UCSC Genome Browser [22] (RefSeq Genes track [23]). Annotations for *Arabidopsis thaliana* and *Saccharomyces cerevisiae* were from TAIR [24] (tair9) and SGD (S288c) [25], respectively. Mitochondrial, chloroplast and unassembled chromosome sets were excluded from further analysis. Multiple splice variants were not considered; in each case, all but one splice variant was discarded. For tair9, the first identified splice variant was retained in the absence of canonical splice information.

From each annotation, we retained the largest set of genes so that there is a unique mapping between the gene identifiers and Uniprot protein identifiers. Feature locations were used to classify regions as intronic, exonic (i.e., coding region), $5^{\prime }$ or $3^{\prime }$ UTR, upstream or intergenic; these mutually exclusive classifications were then used to annotate genomic repeat tracts.

### Genomic repeat tracts

Repeat tracts were identified using Tandem Repeats Finder 4.04 [26], with the following parameters: Match=2, Mis456, match=7, Delta=7, PM=80, PI=10 and Minscore=40. and a maximum repeat period of 3. Identified repeats were further filtered to remove all repeats with a period of one or two; period-one repeats have multiple periodicities, but were here considered to be mono-nucleotide repeats and were excluded from further consideration.

For comparison with other definitions of a repeat, the minimum length under this scoring is $6\frac{2}{3}$ repeat units (i.e., 20 nucleotides) with no mismatches. In subsequent tests involving amino acid tracts (see below), we used a stricter criterion to enable precise reverse mapping from amino acids coding sequence.

### Sequence frequency analysis

For each genome, whole-genome and per-chromosome tri-nucleotide frequencies were determined using a custom script to obtain an order-two Markov background from the whole-genome and chromosome sequences respectively. In order to test whether TNR sequence frequencies were consistent with the (order-two Markov) background, a chi-squared test was used.

### Gene Ontology (GO) associations

GO term associations with genes/gene products were obtained from the Gene Ontology project, as was the ontology itself (version from July 2012). Individual genes or gene products were annotated with each GO term appearing in the association set, and with the transitive closure of those terms. The transitive closure of the GO graph was constructed using only is_a and part_of relationships to avoid false positives (e.g., from has_part or regulates relationships). GO term over-representation was assessed using the Fisher exact test, with Bonferroni correction applied to adjust for multiple hypothesis testing. In preliminary studies, GO-term over-representation in non-coding regions was analysed. The associations discovered were weaker and semantically very similar to those of coding regions, which also have more common disease associations; as a result, non-coding regions were not included in further analyses.

### Proteomic data

The proteomes were downloaded from UniProt, using the UniProt/Swiss-Prot identifiers obtained by mapping gene identifiers from the annotations described above.

Protein-protein interaction data were sourced from the IntAct database [27].

### Amino acid repeat tracts

Repeat tracts were identified by scanning all protein sequences for homo amino acid runs of length at least seven residues for correspondence with the repeat unit thresholds identified by Tandem Repeats Finder. We then examined the coding sequence for each homo-AA tract to determine if it is encoded by a TNR: a homo-AA tract is considered TNR-encoded if at least seven consecutive residues of the tract were encoded by the same codon. (A separate tool XSTREAM [28] is available to identify homo amino acid runs, but due to small discrepancies of what counts as a repeat by Tandem Repeats Finder and XSTREAM we were unable to utilise them to map back and forth between genomic and proteomic repeat locations.) The complete proteome sets were scanned and repeat sequences identified were used as a base set for further study.

### Genomic conservation scores

As a measure of per-site genomic conservation, pre-computed PhastCons [29] scores were used. These were sourced from the UCSC genome browser tables for *Drosophila* (phastCons15way) and human (phastCons46way).

We did not complete this analysis for the other four organisms.

### Tract composition analysis

Analysis of tract composition was undertaken for TNR-encoded homo-AA tracts. The distribution of specific amino-acid repeats encoded by TNRs was assessed by a two-tailed binomial test for each amino acid; success counts were defined as the number of TNR-encoded repeats for that residue, with the probability of success defined as the proportion of TNR-encoded homo-AA proteins over the total set of homo-AA proteins.

Length of homo-AA repeats in TNR- and variant-encoded tracts was compared using the non-parametric Mann-Whitney *U* test. All homo-AA tract lengths were gathered, split into one set per residue, and annotated as being either TNR- or variant-encoded. A significant result indicates that homo-AA repeats tracts of a given amino-acid are longer (or shorter) when TNR-encoded.

## Competing interests

The authors declare that they have no competing interests.

## Authors’ contributions

KW, SB and MB conceived the study. KW, MDC and MB designed and carried out the analyses. JW and SB contributed to data interpretation. KW drafted the manuscript with assistance from MDC, JW, SB and MB. All authors read and approved the final manuscript.

## Supplementary Material

Additional file 1: Figure S1 Distribution of TNR lengths in multiple organisms. The distribution of repeat sequence lengths across different organisms is generally similar. Repeat unit count is logarithmic; frequency is linear, measured as a percentage of the total number of repeat units identified. Click here for file

Additional file 2: Figure S2 Frequency of protein-protein interaction counts for homo-AA proteins. Protein-protein interaction counts for homo-amino acid tract containing proteins in *Saccharomyces cerevisiae*, *Arabidopsis thaliana*, *Caenorhabditis elegans*, *Drosophila melanogaster*, *Mus musculus* and *Homo sapiens*, separated into those that are TNR-encoded and variant-encoded. Whole proteome data is provided as a comparison. Click here for file

Additional file 3: Table S1 Over- and under-represented amino acids in TNR-encoded homo-AA repeats by species. Probabilities that the observed distribution between TNR and variant-encoded amino-acid repeats consisting of specific amino acids is consistent with a random distribution based on overall frequency in *Saccharomyces cerevisiae*, *Arabidopsis thaliana*, *Caenorhabditis elegans*, *Drosophila melanogaster*, *Mus musculus* and *Homo sapiens*. Click here for file

Additional file 4: Table S2 Length comparison of TNR- vs. variant-encoded homo-AA repeats by species. Mean lengths and standard error of given amino-acid repeat sequences in TNR and variant-encoded repeats in *Saccharomyces cerevisiae*, *Arabidopsis thaliana*, *Caenorhabditis elegans*, *Drosophila melanogaster*, *Mus musculus* and *Homo sapiens*. The given p-values represent the probability that the length distributions are equal; these results show that for multiple amino acids, triplet encoded tracts are longer than variant-encoded tracts. Click here for file
