# Supplementary figures and images for: Repeat-encoded poly-Q tracts show statistical commonalities across species
Source: BMC Genomics. 2013 Feb 2;14:76. doi: 10.1186/1471-2164-14-76 (PMC3617014; doi:10.1186/1471-2164-14-76)

Supplementary Figure 1

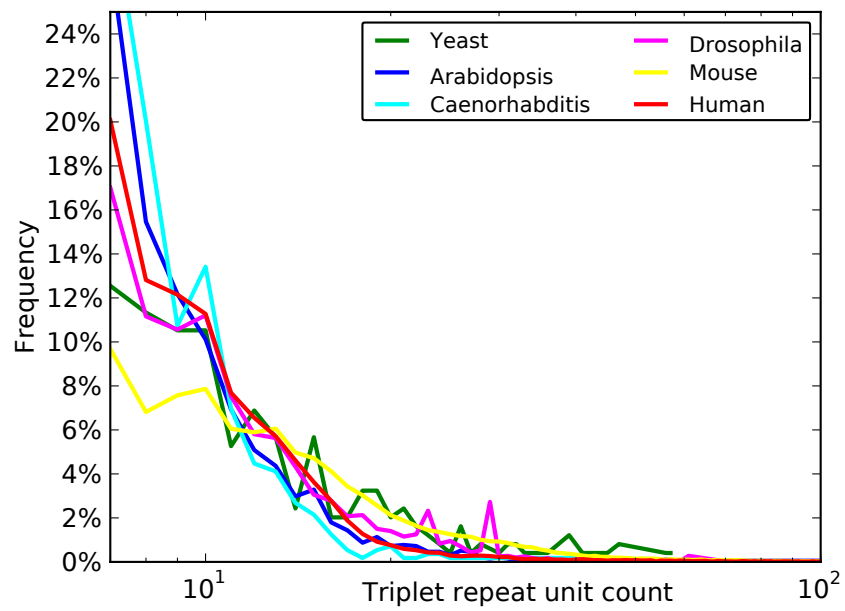

Supplement: Additional file 1: Figure S1 — Distribution of TNR lengths in multiple organisms. The distribution of repeat sequence lengths across different organisms is generally similar. Repeat unit count is logarithmic; frequency is linear, measured as a percentage of the total number of repeat units identified. [file 1471-2164-14-76-S1.pdf]

Supplementary Figure 2

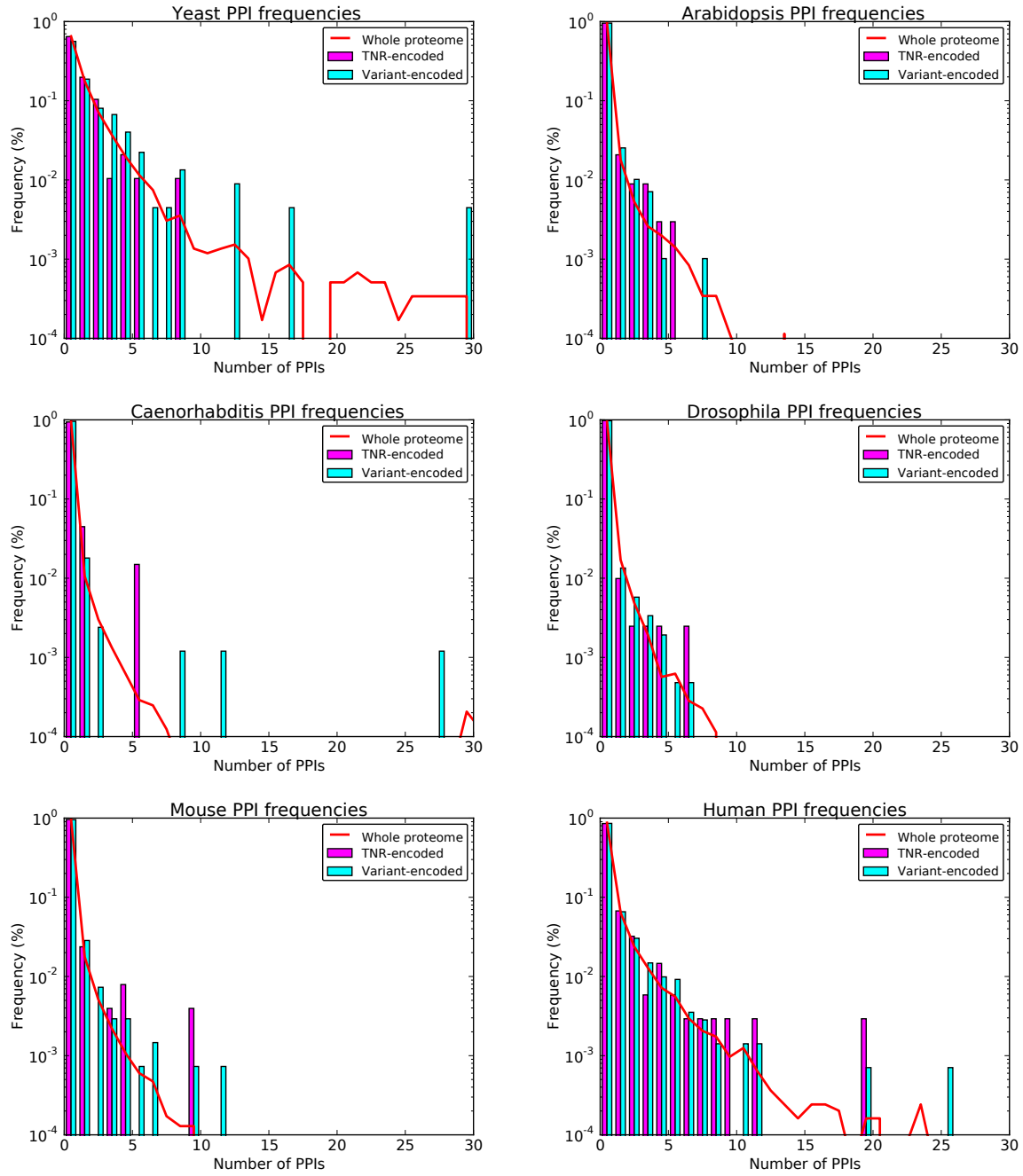

Supplement: Additional file 2: Figure S2 — Frequency of protein-protein interaction counts for homo-AA proteins. Protein-protein interaction counts for homo-amino acid tract containing proteins in Saccharomyces cerevisiae, Arabidopsis thaliana, Caenorhabditis elegans, Drosophila melanogaster, Mus musculus and Homo sapiens, separated into those that are TNR-encoded and variant-encoded. Whole proteome data is provided as a comparison. [file 1471-2164-14-76-S2.pdf]
