# Supplementary material for: Repeat-encoded poly-Q tracts show statistical commonalities across species
Source: BMC Genomics. 2013 Feb 2;14:76. doi: 10.1186/1471-2164-14-76 (PMC3617014; doi:10.1186/1471-2164-14-76)
Supplement: Additional file 3: Table S1 — Over- and under-represented amino acids in TNR-encoded homo-AA repeats by species. Probabilities that the observed distribution between TNR and variant-encoded amino-acid repeats consisting of specific amino acids is consistent with a random distribution based on overall frequency in Saccharomyces cerevisiae, Arabidopsis thaliana, Caenorhabditis elegans, Drosophila melanogaster, Mus musculus and Homo sapiens. [file 1471-2164-14-76-S3.pdf]

Supplementary Table 1

Saccharomyces cerevisiae			*Arabidopsis thaliana*		
Amino acid	E-value	Over/under represented	Amino acid	E-value	Over/under represented
A	6.9		A	0.098	
D	4.3		D	*8.8e-16	over
E	0.74		E	*5e-05	over
G	13		G	*3e-05	under
H	9.6		H	11	
K	5.9		K	1.3	
L	na		L	0.99	
N	*0.0021	over	N	*1.7e-07	over
P	5		P	*0.00011	under
Q	3.3		Q	9.6	
R	13		R	9.3	
S	*0.049	under	S	*0.00017	under
T	13		T	14	

Caenorhabditis elegans			*Drosophila melanogaster*		
Amino acid	E-value	Over/under represented	Amino acid	E-value	Over/under represented
A	2.7		A	*1.1e-09	under
D	12		D	8.2	
E	6.1		E	*0.0055	over
G	12		G	0.49	
H	11		H	10	
K	5.1		K	15	
L	9.3		L	9.2	
N	2		N	*0.035	over
P	5.4		P	*4.5e-06	under
Q	1.6		Q	*1.7e-06	over
R	0.17		R	15	
S	16		S	4.8	
T	0.051		T	*0.015	under

Mus musculus			*Homo sapiens*		
Amino acid	E-value	Over/under represented	Amino acid	E-value	Over/under represented
A	*0.023	under	A	1.3	
D	2.3		D	0.13	
E	2.6		E	0.21	
G	15		G	12	
H	2.8		H	0.96	
K	0.33		K	0.1	
L	*0.038	under	L	1.7	
N	0.31		N	0.46	
P	*1.2e-05	under	P	*5.2e-09	under
Q	*7.9e-35	over	Q	*4.5e-22	over
R	9.7		R	15	
S	13		S	11	
T	10		T	7.8	
