# Supplementary material for: Repeat-encoded poly-Q tracts show statistical commonalities across species
Source: BMC Genomics. 2013 Feb 2;14:76. doi: 10.1186/1471-2164-14-76 (PMC3617014; doi:10.1186/1471-2164-14-76)
Supplement: Additional file 4: Table S2 — Length comparison of TNR- vs. variant-encoded homo-AA repeats by species. Mean lengths and standard error of given amino-acid repeat sequences in TNR and variant-encoded repeats in Saccharomyces cerevisiae, Arabidopsis thaliana, Caenorhabditis elegans, Drosophila melanogaster, Mus musculus and Homo sapiens. The given p-values represent the probability that the length distributions are equal; these results show that for multiple amino acids, triplet encoded tracts are longer than variant-encoded tracts. [file 1471-2164-14-76-S4.pdf]

Supplementary Table 2

Saccharomyces cerevisiae

Amino acid	Triplet length	Variant length	p-value
A	8.50 (0.50)	8.27 (0.24)	4.15e-01
D	13.25 (2.49)	9.19 (0.53)	9.00e-02
E	9.82 (0.98)	8.92 (0.56)	1.08e-01
G	na (na)	7.00 (0.00)	na
H	8.00 (0.00)	8.00 (0.67)	1.82e-01
K	8.00 (0.00)	7.60 (0.60)	9.57e-02
L	na (na)	na (na)	na
N	11.82 (0.85)	10.35 (0.98)	4.70e-02
P	7.50 (0.50)	8.77 (0.43)	1.28e-01
Q	12.62 (1.44)	10.65 (0.44)	1.49e-01
S	14.00 (2.38)	8.63 (0.31)	1.07e-03
T	8.00 (0.00)	7.00 (0.00)	na

Arabidopsis thaliana

Amino acid	Triplet length	Variant length	p-value
A	7.00 (0.00)	8.17 (0.32)	1.06e-01
D	9.97 (0.68)	8.12 (0.26)	2.11e-02
E	10.42 (0.88)	7.90 (0.12)	8.98e-05
G	9.17 (0.95)	8.35 (0.15)	1.32e-01
H	8.62 (0.80)	8.64 (0.56)	4.70e-01
K	7.84 (0.27)	7.53 (0.17)	1.37e-01
L	7.00 (0.00)	7.42 (0.23)	na
N	9.52 (0.56)	7.30 (0.12)	3.06e-04
P	9.33 (0.49)	8.44 (0.18)	2.26e-02
Q	9.91 (0.71)	8.73 (0.25)	7.13e-02
S	8.74 (0.21)	8.20 (0.10)	1.34e-04
T	7.60 (0.40)	8.30 (0.29)	7.06e-02

Caenorhabditis elegans

Amino acid	Triplet length	Variant length	p-value
A	8.12 (0.44)	7.95 (0.18)	1.90e-01
D	9.00 (1.00)	7.81 (0.12)	9.64e-02
E	11.00 (1.34)	7.78 (0.24)	3.75e-04
G	9.57 (1.19)	7.85 (0.11)	8.94e-02
H	7.50 (0.50)	7.29 (0.11)	2.32e-01
K	10.00 (3.00)	7.59 (0.17)	2.79e-01
L	7.00 (0.00)	7.77 (0.26)	na
N	8.25 (0.95)	7.96 (0.24)	4.88e-01
P	8.14 (0.55)	8.26 (0.15)	4.50e-01
Q	8.73 (0.57)	8.12 (0.11)	2.32e-01
S	8.00 (0.17)	8.90 (0.29)	2.83e-01
T	8.25 (0.75)	8.63 (0.15)	4.69e-01

Drosophila melanogaster

Amino acid	Triplet length	Variant length	p-value
A	9.65 (0.52)	8.99 (0.08)	2.16e-01
D	9.38 (0.91)	7.95 (0.18)	5.77e-02
E	7.69 (0.24)	9.83 (0.94)	1.98e-01
G	10.71 (0.60)	8.56 (0.11)	4.69e-05
H	7.73 (0.25)	8.62 (0.18)	3.54e-02
K	7.00 (0.00)	7.18 (0.10)	na
L	na (na)	7.40 (0.27)	na
N	10.43 (0.60)	8.64 (0.13)	3.39e-04
P	9.00 (1.68)	8.35 (0.15)	4.42e-01
Q	10.43 (0.26)	9.71 (0.09)	7.16e-04
S	9.17 (0.40)	8.85 (0.16)	1.83e-02
T	8.89 (0.46)	9.82 (0.31)	2.39e-01

Mus musculus

Amino acid	Triplet length	Variant length	p-value
A	9.68 (0.62)	9.41 (0.16)	4.49e-01
D	8.67 (0.67)	8.85 (0.38)	2.72e-01
E	11.20 (0.73)	9.35 (0.16)	1.48e-03
G	11.42 (0.76)	8.87 (0.21)	9.65e-05
H	13.75 (3.15)	9.23 (0.28)	2.33e-02
K	8.33 (1.33)	7.78 (0.11)	4.48e-01
L	9.43 (0.65)	7.67 (0.09)	2.10e-04
N	8.00 (0.00)	na (na)	na
P	9.95 (0.75)	9.07 (0.14)	1.33e-01
Q	11.87 (0.54)	11.50 (0.58)	4.35e-02
S	12.32 (1.43)	10.39 (0.42)	5.48e-02
T	11.17 (0.79)	8.03 (0.19)	5.72e-04

Homo sapiens

Amino acid	Triplet length	Variant length	p-value
A	10.98 (0.50)	9.16 (0.15)	5.43e-05
D	10.62 (0.94)	7.67 (0.19)	3.86e-03
E	9.81 (0.41)	9.11 (0.16)	2.04e-02
G	10.54 (0.65)	8.74 (0.18)	3.21e-04
H	9.36 (0.49)	8.69 (0.17)	2.49e-01
K	9.25 (0.48)	7.82 (0.09)	3.00e-03
L	8.44 (0.22)	7.68 (0.09)	1.71e-04
N	7.50 (0.50)	na (na)	na
P	11.05 (1.04)	8.48 (0.12)	1.43e-03
Q	15.28 (0.78)	10.41 (0.66)	4.00e-10
S	14.50 (1.94)	9.55 (0.29)	2.05e-03
T	9.75 (1.38)	8.00 (0.41)	1.15e-01
